# A theoretical model of health management using data-driven decision-making: the future of precision medicine and health

**DOI:** 10.1186/s12967-021-02714-8

**Published:** 2021-02-15

**Authors:** Eva Kriegova, Milos Kudelka, Martin Radvansky, Jiri Gallo

**Affiliations:** 1 Department of Immunology, Faculty of Medicine and Dentistry, Palacky University Olomouc & University Hospital Olomouc, Hnevotinska 3, 775 15 Olomouc, Czech Republic; 2 Department of Computer Science, Faculty of Electrical Engineering and Computer Science, VSB-Technical University of Ostrava, 17. listopadu 2175/15, Poruba, 708 00 Ostrava, Czech Republic; 3 Department of Orthopedics, Faculty of Medicine and Dentistry, Palacky University Olomouc, Hnevotinska 3, 775 15 Olomouc, Czech Republic; 4 Department of Orthopedics, University Hospital Olomouc, I. P. Pavlova 6, 779 00 Olomouc, Czech Republic

**Keywords:** Precision medicine, Precision health, Electronic health record, Clinical decision-making tool, Health trajectory, Early reoperation, Revision rate, Total knee arthroplasty, Lifestyle factors, Formal concept analysis

## Abstract

**Background:**

The burden of chronic and societal diseases is affected by many risk factors that can change over time. The minimalisation of disease-associated risk factors may contribute to long-term health. Therefore, new data-driven health management should be used in clinical decision-making in order to minimise future individual risks of disease and adverse health effects.

**Methods:**

We aimed to develop a health trajectories (HT) management methodology based on electronic health records (EHR) and analysing overlapping groups of patients who share a similar risk of developing a particular disease or experiencing specific adverse health effects. Formal concept analysis (FCA) was applied to identify and visualise overlapping patient groups, as well as for decision-making. To demonstrate its capabilities, the theoretical model presented uses genuine data from a local total knee arthroplasty (TKA) register (a total of 1885 patients) and shows the influence of step by step changes in five lifestyle factors (BMI, smoking, activity, sports and long-distance walking) on the risk of early reoperation after TKA.

**Results:**

The theoretical model of HT management demonstrates the potential of using EHR data to make data-driven recommendations to support both patients’ and physicians’ decision-making. The model example developed from the TKA register acts as a clinical decision-making tool, built to show surgeons and patients the likelihood of early reoperation after TKA and how the likelihood changes when factors are modified. The presented data-driven tool suits an individualised approach to health management because it quantifies the impact of various combinations of factors on the early reoperation rate after TKA and shows alternative combinations of factors that may change the reoperation risk.

**Conclusion:**

This theoretical model introduces future HT management as an understandable way of conceiving patients’ futures with a view to positively (or negatively) changing their behaviour. The model’s ability to influence beneficial health care decision-making to improve patient outcomes should be proved using various real-world data from EHR datasets.

## Background

Long-term health is a delicate combination of nutrition, lifestyle, environment and genetics, dedication to maintaining and improving one’s health, and the assiduous avoidance of health-damaging behaviours. A health trajectory (HT) is a useful way of portraying the dynamic course of health and disease and presents an individual’s health as a factor dependent on time. The risks for a particular disease are influenced by many factors, which may change in specific situations over time [1, 2]. Nowadays, many risk factors, as well as other health and/or disease-related data, are detailed in a hospital or outpatient electronic health records (EHR) [3–6]. Increasingly, patients are willing to share more and better data with the health care system. Therefore, there is an urgent need to develop a process of automated analysis for this data, which could result in establishing a clinical decision-making tool (CDMT) as a component of a clinical decision support system (CDSS), which in turn, ultimately leads to the reduction of the individual risks associated with certain diseases or adverse health effects [7–9].

The quality of decision-making in the era of precision health and precision medicine (see description of these terms below) is influenced by three groups of data that are related to time. The first group is data describing the patient’s current condition reported as a set of factors in their EHR. The second group is data representing the patient’s history, which is (or should be) included in the EHR, such as the patient’s initial condition and its changes over the time preceding their current condition. The third group of data is related to a description of the patient’s specific living conditions and their future changes, which are not included in the EHR.

Based on the huge amount of data available in EHRs, including hundreds of demographic, laboratory and clinical factors, there is an urgent need to develop computational approaches and CDMTs based on combinations of patient factors to support decision-making about effective health and disease management [10, 11]. These approaches should allow the clinician(s) and patient(s) to evaluate together the qualitative and quantitative contributions of numerous factors to the medical risk, such as the disease, treatment response, failure, complication and/or prognosis in individual patients, as already shown in real-world cohorts [12–14]. Additionally, patients could be informed about the impact of particular factors on the likely outcome. The CDMT would allow decision-making, shared between patients and clinicians, to be based on intelligible recommendations. Finally, this approach might modify patients’ expectations, which is a factor strongly affecting not only future care or interventions [11], but also their outcomes. Nevertheless, there is a lack of computational approaches that could quantify the contribution of risk factors on health [15, 16].

We aimed to develop an HT management strategy that could identify and utilise factors that can affect, individually or in combination, an individual’s future health. The introduced theoretical model was presented using the clinical registry dataset presented in our previous study [17], revealing the positive effect of five lifestyle factors (normal BMI, non-smoking, activity, sports and long-distance walking) on reducing the risk of early reoperation after total knee arthroplasty (TKA). HT management based on continuous data-driven decision-making is a long-term strategy to manage health, irrespective of the branch of medicine.

## Material and methods

### HT management

Working with a large amount of patient data in the form of EHRs (all the information collected and archived in hospital or outpatient electronic databases, including registries for particular treatments) and using automated processing and analysis methods based on machine learning or, generally, on artificial intelligence should result in CDMTs that can intelligently support clinicians’ and patients’ decision-making [11]. As mentioned in the introduction, our approach focuses on the patient’s influenceable future, starting with their initial condition and history saved in the EHR. The patient’s future is understood as a risk (or set of risks) of disease and adverse health effects. Their options for reducing the risk are then analysed based on the factors that probably influence their risk.

Four assumptions have been made as follows:

1. The patient can influence their individual factors (or at least some of them).
2. Each combination of selected factors defines a group of patients as similar in terms of these factors, and they are exposed to a similar risk level.
3. The degree of risk of developing a disease or medical condition can be quantified for each combination of values of the selected factors. Combinations of factors may overlap, or one combination may be part of other larger combinations.
4. The more specific the combination of factors considered, the smaller the group of patients. Individual groups then differ in their degree of risk. Because combinations of factors may overlap, groups of patients may also overlap, or an even more specific group of patients may be included in a less specific larger group of patients. This is the most important assumption; it is a consequence of the previous three assumptions and is related to the factors that will be examined.

These assumptions underpin the logic of which groups the patient belongs to. At the same time, thanks to a change in the factors examined and influenced by the patients, they can move to another group with a lower (or higher) level of risk. Due to the complex relationships between groups, there are always more options for moving to a group with less (or higher) risk. Moreover, the move towards a lower risk may or may not be a one-off. On the contrary, it is assumed that it will be repeated over time with a clinician’s or physiotherapist’s possible supervision. This creates a process called ‘health trajectory management’. The individual steps of this process performed over time create a trajectory leading, ideally, to a continuous reduction of risk and/or an improvement in health characteristics. Implementing these factors on the particular patient(s) can affect the outcomes of therapeutic interventions, at least in part, via the reduction of harm associated with an intervention. Therefore, all stakeholders (patients, their physicians, clinical settings and insurance systems) may profit from such approaches.

Although this is a task that is generally very complex in scope and content, the essence of HT management can be shown in a simple and understandable example, which will be given below. This example is based on the results of previously published research [17], which showed the positive effect of five factors (normal BMI, non-smoking, activity, sports and long-distance walking) on reducing the risk of early TKA reoperation. For this study on HT management, a dataset with familiar patients was used where their conditions were known before undergoing TKA surgery, and whether they underwent early reoperation and the five factors were binarised (for example, non-smokers/ex-smokers = zero, smokers = one).

As mentioned above, an essential requirement for the analysis is that there can be overlapping groups of patients characterised by agreement in the factors studied. A traditional method that can detect overlapping combinations of binary factors and overlapping groups is formal concept analysis (FCA), which results in a visual structure describing overlapping groups (clusters), the so-called ‘concept lattice’ [18–20]. The use of binary factors and a concept lattice may seem to be limiting factors in this approach, but this is not the case. The concept lattice offers a simple introduction to HT management. For non-binary factors, it is possible to use one of the overlapping clustering methods for the same purpose [21, 22].

HT management is not focused on a one-time prediction: its usual goal is to place the patient into a group with common characteristics and subsequent treatment. The purpose of HT management is to interact with the explorative interface of CDMT repeatedly and move patients who, in terms of their condition and history, belong to one group into one of the more specific groups with less risk. HT management targets factors that can be affected either by the patient or their physicians and that, individually or in combination, can positively or negatively influence the patient’s future condition.

### Analytical model: grouping patients into overlapping clusters

FCA and the concept lattice are used to illustrate our approach (see Additional file 1: Tables S1 and S2 and Figure S1). First, patient groups are formed into a concept lattice using FCA [23–25] applied to a selected set of binary factors. In this real-world example, there were five preoperative lifestyle factors. The formal concepts are clusters that indicate relationships hidden in the data among patients with a common subset of lifestyle factors. Concepts are derived from the table containing patient factors called ‘context’ [26]. By ordering concepts, a mathematical structure called a concept lattice is obtained that describes relationships between individual groups (concepts) of patients with a shared set of factors. Such a structure enables the visualisation of the concepts in hierarchical form. For example, the concept can be a group of patients who are non-smokers and have a BMI < 30, regardless of other factors. When physical activity is added to these two factors, a new, smaller (and more specific) concept containing a group of non-smoking patients with a BMI < 30 who also participate in physical activity will be formed.

Thus, we can create a sequence in which the first concept contains the most patients, and the last contains the least patients. The smaller the concept, the more specific it is and the more similar the patients are. Each of these concepts (clusters) carries a different likelihood of risk.

### Quantifying contributions of changes in modifiable clinical factors

If the investigated factors are modifiable, then a sequence of concepts could be identified in which (i) the factors in the preceding concept are also contained in the succeeding concept of the sequence, (ii) patients in the succeeding concept are contained in the preceding concept of the sequence and (iii) the likelihood of risk in the succeeding concept is lower than in the preceding concept. All such sequences can then be understood as possible HTs because they continually reduce the risk’s likelihood. In each of these trajectories, the first cluster describes the patient’s current condition, and in subsequent clusters of the trajectory, the number of patients decreases and the modifiable factors that contribute to the expected outcomes in the future increase. On the other hand, if the patient’s condition changes to the previous cluster of the trajectory, their risk’s likelihood increases.

That said, some concepts can contain very few patients and zero risk events. The empirical probability of reoperation is zero in these cases. To reflect that reoperation may also occur in these small groups, some uncertainty was introduced into the dataset before further analysis was performed (for more details, see the Additional file 1). This slightly changed the empirical probability. The modification did not change the reoperation probability for the entire dataset. For a higher empirical probability than total, reoperation probability decreases, and for a lower empirical probability than total, reoperation probability increases for the individual concepts. This is also true for the zero value that also increased the probability.

### Visualisation of HT trajectories

Concepts for each patient subgroup and the relationships between them can be visualised as a weighted-directed network. The vertices (circles) of the network represent individual concepts. The directed edges (arrows) represent whether the risk of reoperation increased or decreased after adding a factor. The groups of concepts connected in a sequence by arrows represent HT with gradually added factors that decrease the risk of reoperation.

The size of the vertices (concepts) corresponds to the risk of reoperation. The same holds for vertex labels with factors. The edge (arrow) strength and its label correspond to the reduction of reoperation risk after adding a positive factor (indicating how much the risk of reoperation would be reduced). On the other hand, removing a positive factor may be understood as adding a negative factor, leading to a higher risk of reoperation. The colours of the vertices and edges indicate the reliability of the estimation. Concepts (vertices) containing at least ten percent of patients are green, concepts that have an original empirical probability equal to zero are red, and other concepts are yellow. The ten percent threshold was selected based on the size of the dataset to draw attention to the lower reliability of the recommendations when examining visualised HT and interacting with a CDMT.

### Health trajectory example

**Fig. 1:**
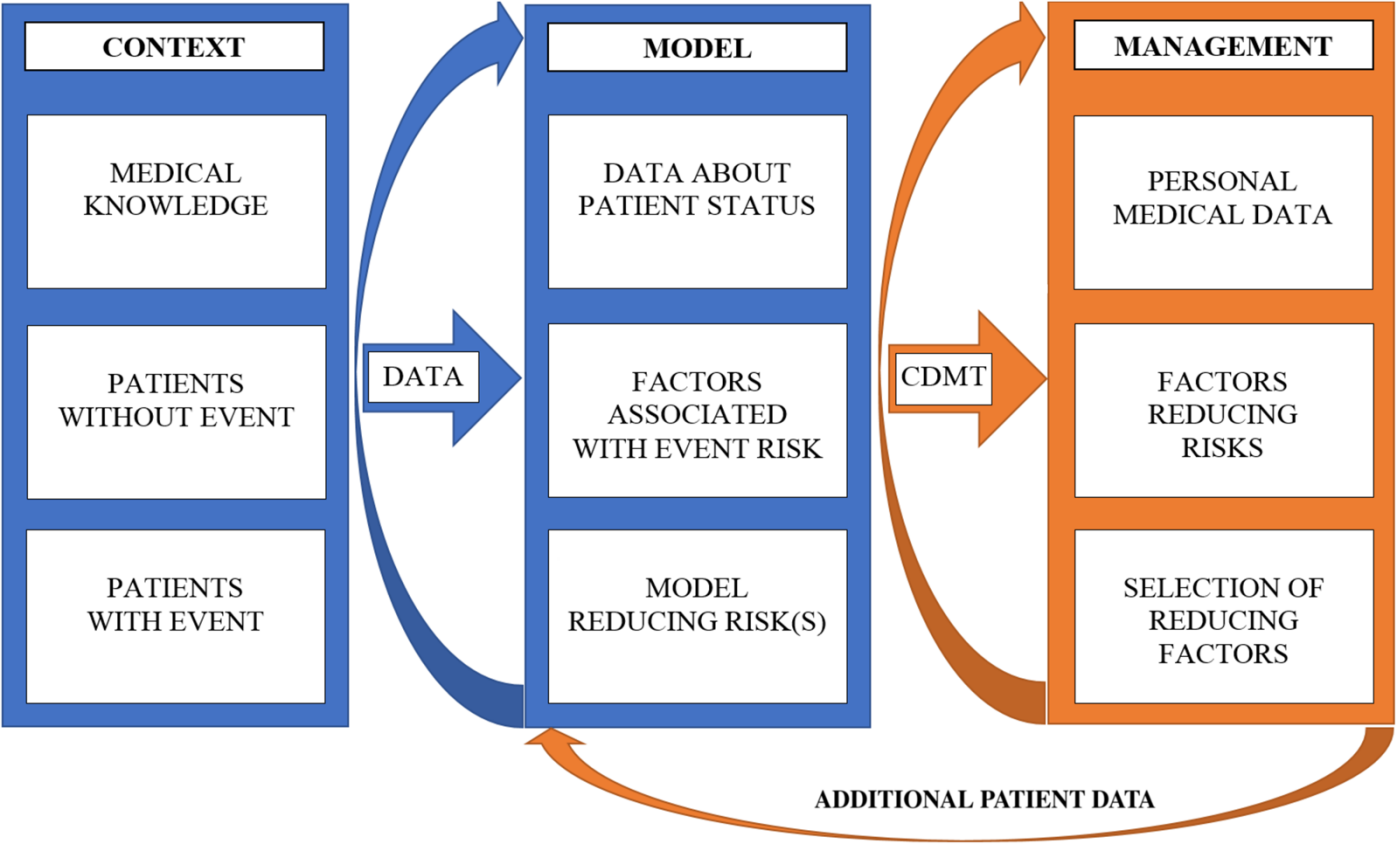
Scheme of general health trajectory (HT) management. HT management consists of three steps: (1) context leading to the definition of a medical problem and risk event, acquisition and evaluation of patient data; (2) an analytical data model based on data analysis, analysis of factors associated with risk events, identification of risk factors associated with risk events and a data model for a CDMT; and (3) CDMT for patient management based on a patient’s personal characteristics. Newly generated patient data can enter the data modelling step, refining the assessment of the likelihood of a medical event

Our approach was applied to a real-world cohort of patients with TKA, and the contribution of modifiable lifestyle factors to the risk of reoperation was evaluated. Our methodology of HT management consists of three components: (i) context, leading to the definition of a medical problem and risk event, acquisition and evaluation of patient data, (ii) an analytical data model identifying risk factors and possible HTs, and (iii) implementing the model in a CDMT and providing a user interface to support clinical decision-making (see Fig. 1).

### Dataset (patient cohort)

To present our model, an unselected real-world cohort of 1885 patients (695 men and 1190 women) who underwent TKA surgery between September 2010 and April 2017 at a single tertiary orthopaedic centre was analysed. For all patients, the lifestyle and clinical factors before TKA surgery, as well as information regarding early reoperation (defined as less than two years after primary surgery), were available in the clinical register. Based on different reoperation rates in younger and older patients, subgroups were created based on the median number of reoperations in the male and female groups (younger females ≤ 71 years, older females > 71 years; younger males ≤ 66 years, older males > 66 years), respectively [17]. For clinical and lifestyle factors in the enrolled patients and gender and age subgroups, see Table 1 and Additional file 1: Table S3.

**Table 1:** Demographic and lifestyle parameters in the TKA patient cohort

Parameters	Value	Younger females (≤ 71 years) N = 670		Older females (> 71 years) N = 520		Younger males (≤ 66 years) N = 275		Older males (> 66 years) N = 420	
		N	%	N	%	N	%	N	%
BMI [kg/m^2^]	< 30	235	35.1	261	50.2	116	42.1	237	56.4
	31–35	226	33.7	178	34.2	89	32.4	147	35.0
	> 35	209	31.2	81	15.6	70	25.5	36	8.6
Smoking	No	538	80.3	479	92.1	166	60.4	298	71
	Stop	56	8.4	23	4.4	56	20.4	90	21.4
	Yes	76	11.3	18	3.5	53	19.3	32	7.6
UCLA activity	No/low (1–3)	545	81.3	460	88.5	181	65.8	325	77.4
	Middle (4–6)	125	18.7	60	11.5	94^a^	34.2	95	22.6
Sport activity	No	607	90.6	483	92.9	216	78.5	342	81.4
	Active	43	6.4	24	4.7	49	17.8	56	13.3
	NA	20	3.0	13	2.5	10	3.6	22	5.2
Reoperation	No	643	96.0	495	95.2	245	89.1	393	93.6
	Yes	27	4.0	25	4.8	30	10.9	27	6.4

TKA: total knee arthroplasty; BMI: body mass index; UCLA: University of California Los Angeles; NA: not available.

^a^one patient with UCLA high (7–10) included

### Investigated lifestyle factors

To demonstrate the capabilities of our model, the following preoperative factors were included: physical activity, sports activity, smoking, body mass index (BMI) and the ability to walk long distance (1000 m). Physical activity was evaluated using the University of California Los Angeles (UCLA) activity scale [27]. In terms of UCLA, an inactive patient was one who reported no or low physical activity (categories one to three). An active patient (categories four to six) reported regular participation in mild (walking) or moderate activities, such as swimming, unlimited housework or shopping. A high degree of activity was defined as categories seven to ten, according to UCLA. Sports activity was evaluated based on the patients’ subjective estimations of their participation in sport, distinguishing between none, recreational, competitive and professional performance levels. A BMI (calculated as weight in kilograms divided by height in square metres) of 30 of over was considered obese (obesity I: BMI 30–35; obesity II: BMI > 35).

The individual factors were binarised as (i) no physical activity (UCLA categories ≤ four) versus physical activity (UCLA categories > four, performing unlimited housework and shopping), (ii) no sports activity versus sports activity (recreational, competitive and professional performance levels), (iii) smoking versus non-smoking (including ex-smokers) and (iv) normal/overweight (BMI < 30) versus obese (BMI ≥ 30).

## Results

### Observed concepts in males and females

Table 1 shows the demographic and lifestyle factors of a real-world patient cohort with TKA from the clinical register of joint replacements used for testing our approach. The concepts were calculated for younger and older females (see Additional file 1: Tables S4 and S5), and younger and older males (see Additional file 1: Tables S6 and S7) separately as other factors influence the rate of reoperations in each patient subgroup. The sequences of concepts associated with reducing the likelihood of reoperation in TKA patient subgroups are shown in Additional file 1: Figure S2. For each concept, the number of patients in the concept, the number of patients who underwent early reoperation, the percentage of probabilities, including the empirical probability of reoperation in the concept, and the given uncertainty are presented.

As an example, observed concepts in older women will be discussed (Additional file 1: Table S5). The first row of Additional file 1: Table S5 is a concept containing older women with no common factors. The percentage of reoperations in this concept (and, thus, the total proportion of reoperations among older women) is 4.98%. The next rows show the percentage of reoperations in each subgroup defined by combinations of factors. For example, adding the activity factor, regardless of BMI, smoking, sport and long-distance walking, produces a smaller group with 11.75% of older women and more precise information on the reoperation rate (3.39%).

### Data analysis and outputs of the CDMT

To obtain information about the risk for early reoperation in a patient before the primary TKA, a CDMT was developed using an appropriately structured and validated dataset. In step one, the patient types their gender and age into the CDMT. The overall reoperation rate in patients within that gender and age group will be presented based on real-world data from a registry of total joint arthroplasty. In step two, the patient selects their preoperative factors: non-smoking status (Y/N), activity (Y/N), ability to walk 1000 m (Y/N) and sports activity (Y/N). The CDMT shows the percentage of patients with the same preoperative factors for TKA, and the reoperation rate in this patient group based on the registry data. In a further step, the patient could add individual factors that they wish to change prior to the primary surgery, and the CDMT calculates the size of the group and the reoperation rates based on the corresponding group of patients with those factors. The CDMT can provide the patient with information about how to positively change their level of risk and promote confidence in taking that step. After testing the impact of individual factors, combinations could be tested. The patient may choose to modify the factors: for example, those that result in the lowest reoperation rates and/or those that they can influence themselves.

To show the practical output of our CDMT, examples of two TKA patients will be presented: a non-smoking older woman and an older man who smokes. The CDMT shows the best combinations of positive factors, as well as the order of changes needed to achieve the best outcome (the lowest risk for early reoperation). To gain full insight into the calculations, all the data is shown, even when the difference in the likelihood of reoperation by changing a particular factor or their combinations is relatively small. Nevertheless, even small changes may move the particular patient into the group with better or worse outcomes.

*Case study A***:** Woman, 78 years old, non-smoker, no activity (limited housework, no shopping), no long-distance walking, a BMI of 36, no sports activity.

**Fig. 2:**
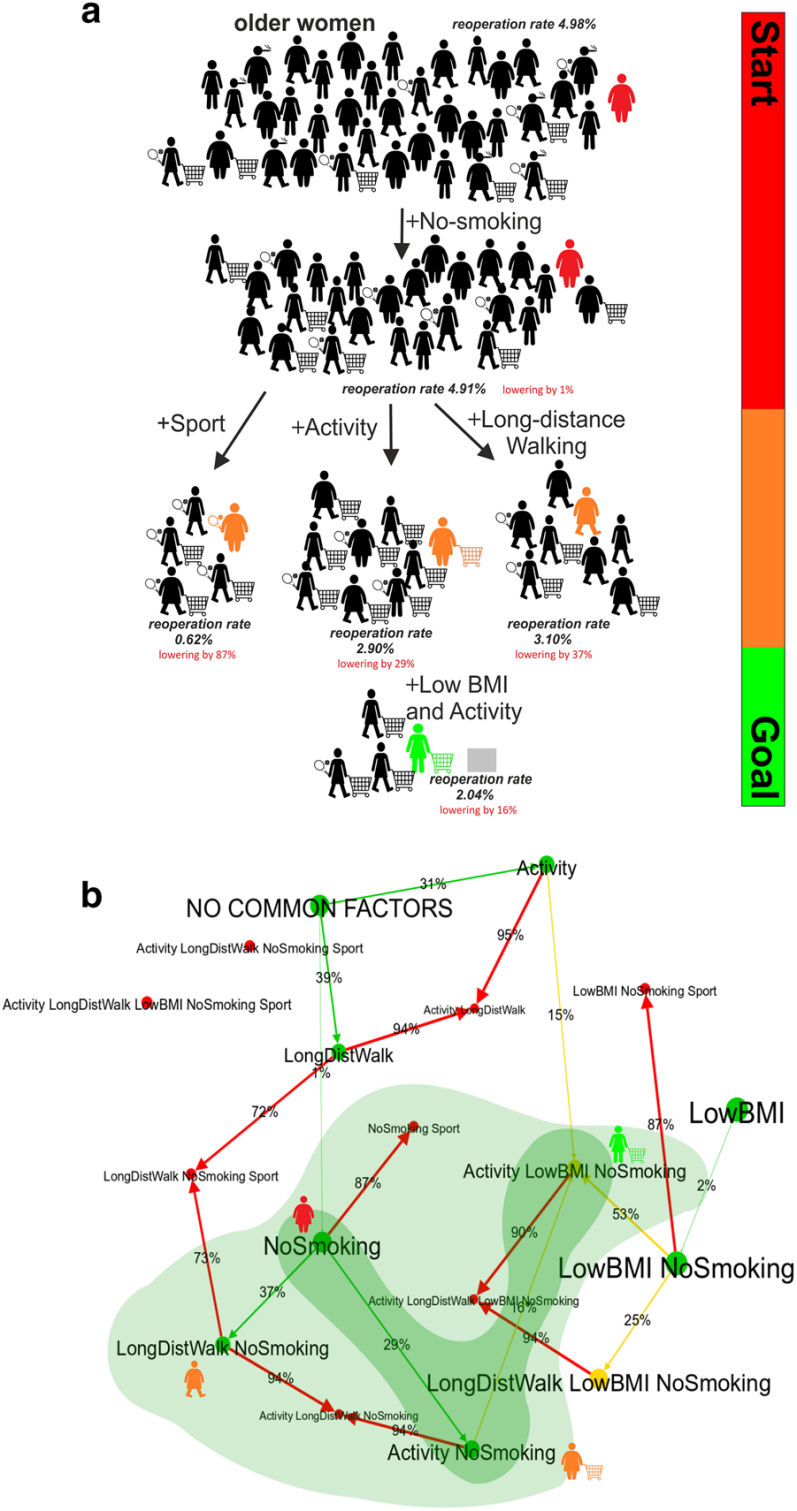
Sequence of concepts associated with reducing the likelihood of reoperation in a particular woman (shown in colour: 78 years old, non-smoker, not active, no long-distance walking, BMI of 36, no sports activity). A representative example of a CDMT based on real-world data. The edge (arrow) strength and its label correspond to the reduction of the risk of reoperation after adding a factor (percentage of how much the risk of reoperation would be reduced). The same holds for the vertex labels with factors and the numbers of patients. Methods of reducing the likelihood of reoperation in this specific case are coloured light green, and the most effective method is shown in dark green. Positive factors were activity (Activity), long-distance walking (LongDistWalk), no smoking (NoSmoking), a BMI < 30 (lowBMI) and no positive factors present (NO COMMON FACTORS). The colour of the presented case changes (from red to orange then green) as the probability of reoperation decreases

The revision rate in the whole group of older women is 4.98% (see Fig. 2 and Additional file 1: Table S5). In this group, only 11% of women were physically active using UCLA’s classification, 7% reported sports activity, 14% could walk 1000 m, about 50% had a BMI of below or equal 30, and 92% were non-smokers. The sequences of concepts associated with reducing the likelihood of reoperation in older women are shown in Fig. 2.

**Fig. 3:**
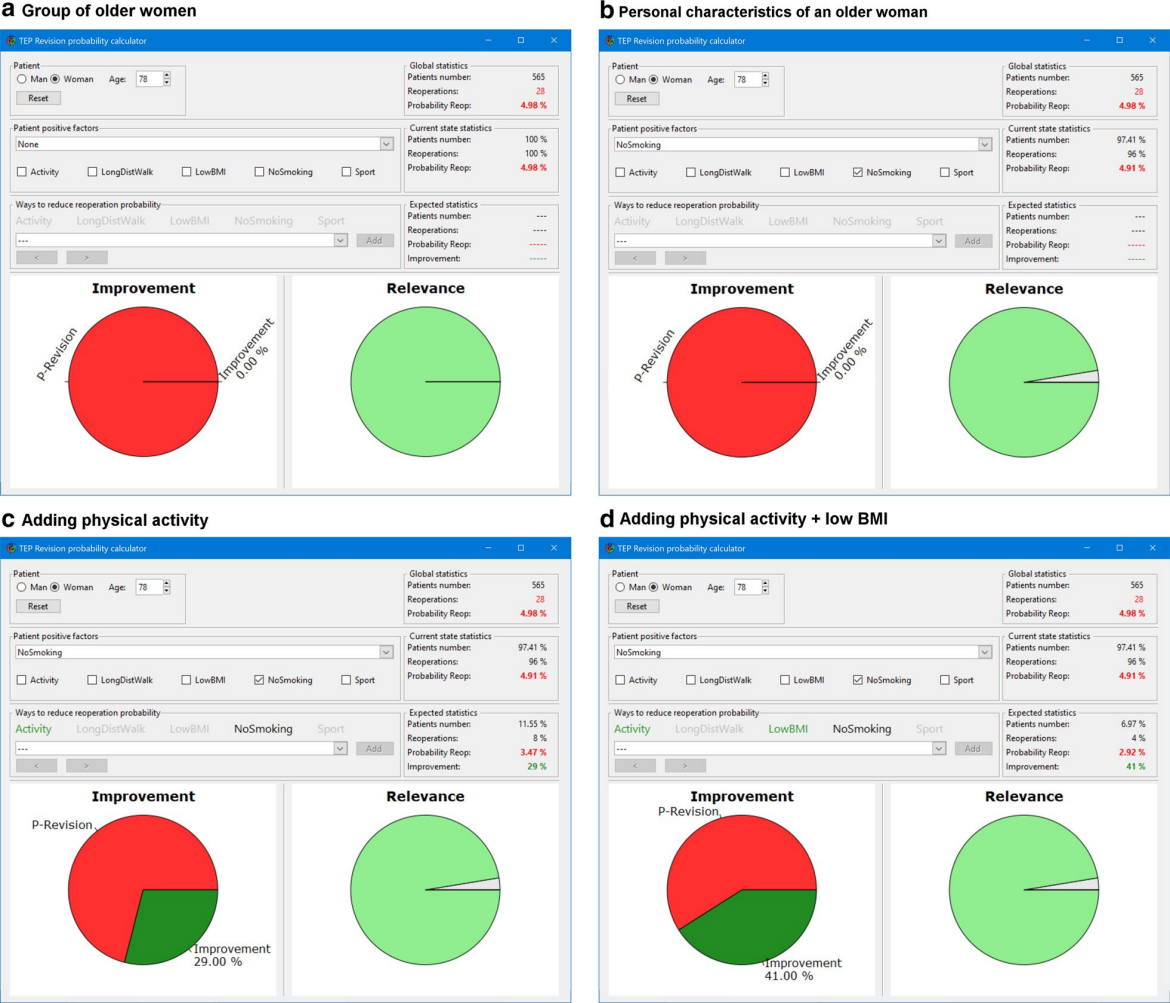
The output of the clinical decision-making tool (CDMT) for the older woman (78 years old, a BMI of 36, no activity, no sport, non-smoking)–a representative example. The screens show **a** the revision rate in the whole group of older women; **b** the likelihood of revision rate in a particular older woman, based on her lifestyle parameters; **c** the likelihood of revision rate and improvements after adding physical activity for this particular woman (reduction of the likelihood of reoperation by 29%); **d** the likelihood of revision rate and improvements after adding physical activity + BMI < 30 for this particular woman (likelihood of reoperation reduced by 45%)

After adding non-smoking, which is the only preoperative factor reducing the likelihood of reoperation for this particular woman, the CDMT calculates the probability of a revision rate of 4.91%. After including another individual positive factor or a combination of factors for this woman, the CDMT calculates the likelihood of reoperation and corresponding improvement when those factors are modified (Fig. 3).

For this woman, there are three suggested ways to reduce the likelihood of reoperation. First, when adding sports activity, the likelihood of reoperation lowers by 87% to a revision rate of 0.62%. However, obese people with no or low physical activity cannot suddenly be expected to start sports activity prior to TKA surgery. This would not be feasible for this particular woman. The second is to add long-distance walking (1000 m), which may lower the probability of reoperation by 37% (to a revision rate of 3.10%). However, it may be difficult to start long-distance walking in the case of a woman with a severe osteoarthritic knee and no to low activity (see Fig. 2). The third way seems to be the most feasible for this particular women: she may start with physical activity in the form of unlimited housework and shopping, which will lower the probability of reoperation by 29% (to a revision rate of 2.90%). If this is followed by lowering her BMI, the combination of these factors may further decrease the probability of reoperation by 16% (to a revision rate of 2.04%). For a female patient with a BMI < 30, who follows these recommendations and becomes active, the revision rate reduces to 0.15% by including long-distance walking.

*Case study B*: Man, 75 years old, smoker, no activity, a BMI of 33, no sports activity.

**Fig. 4:**
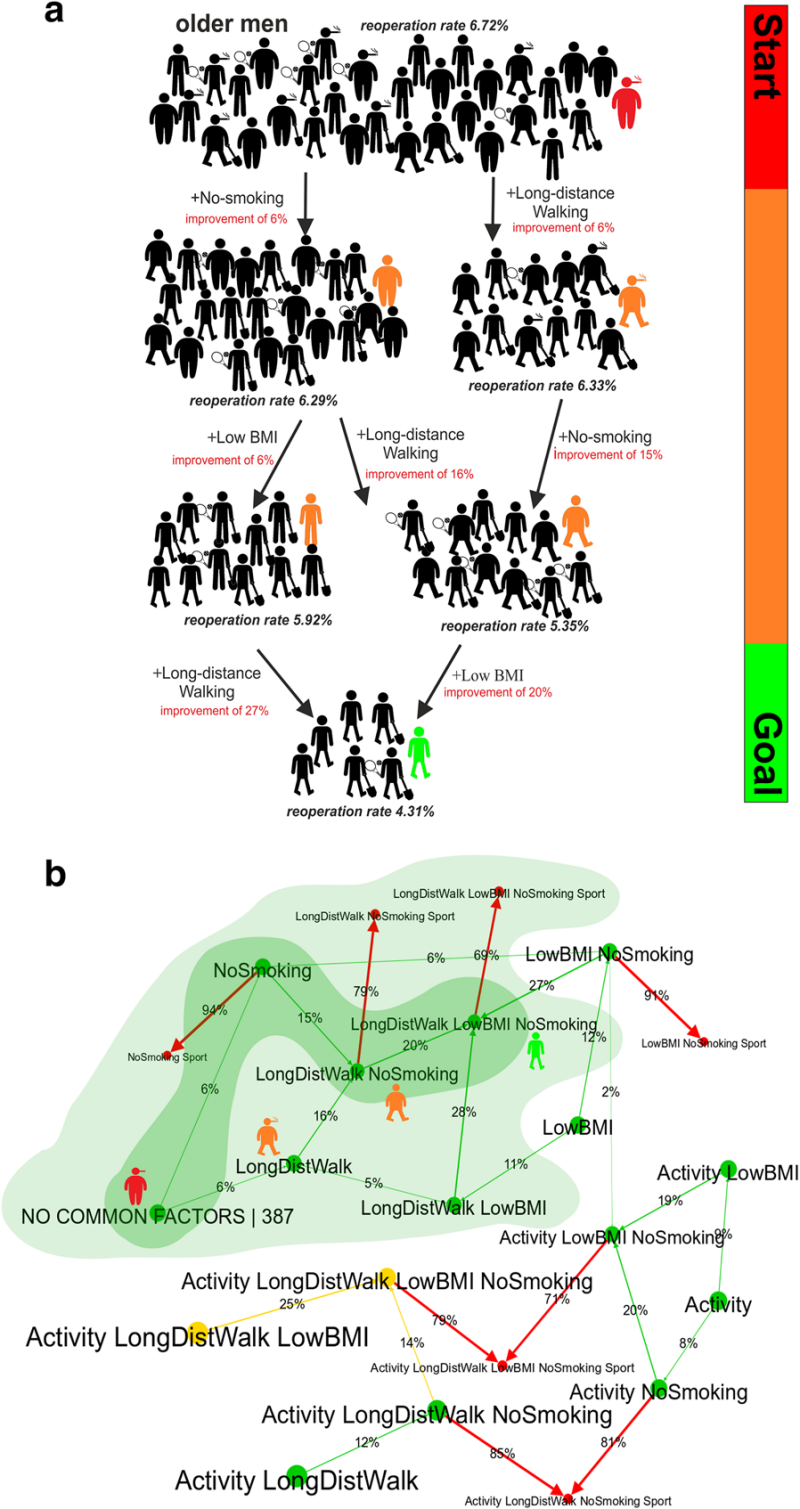
Concepts associated with reducing the likelihood of reoperation in a particular man (shown in colour: 75 years old, smoker, not active, no long-distance walking, BMI of 33). A representative example of a CDMT based on real-world data. Men and women are expected to undertake different physical activities. The edge (arrow) strength and its label correspond to the reduction of the risk of reoperation after adding a factor (percentage of how much the risk of reoperation would be reduced). The same holds for vertex labels with factors and the numbers of patients. Methods of reducing the likelihood of reoperation in this specific case are coloured light green, and the most effective method is shown in dark green. Positive factors were activity (Activity), long-distance walking (LongDistWalk), no smoking (NoSmoking), a BMI < 30 (lowBMI) and no positive factors present (NO COMMON FACTORS). The colour of the presented case changes (from red to orange then green) as the probability of reoperation decreases

In the group of older men, only 23% were physically active in terms of UCLA’s classification, 19% reported sports activity, 22% could walk 1000 m, about 50% had a BMI of below 30, and 71% were non-smokers. This older, obese man (smoker, no physical activity, no sports activity) has no preoperative factors reducing the likelihood of reoperation, meaning the probability of reoperation is 6.72% (see Fig. 4). For this man, there are three suggested ways to preoperatively reduce the likelihood of reoperation: adding no-smoking, long-distance walking (1000 m) and lowering his BMI via a diet or operatively (see Fig. 4). Step by step, the best method for this man to improve his chances of avoiding reoperation are to stop smoking (improvement of 6%), then starting to walk longer distances (improvement of 16%) followed by lowering his BMI (improvement of 20%). By following these steps, the man’s likelihood of reoperation is reduced to 4.31% (see Fig. 4).

Additionally, the model can also visualise what happens if a negative factor is added. Take, for example, a 75-year-old man indicated for TKA. He is an ex-smoker, who does no activity or sports activity with a BMI of 33 who starts smoking. By starting smoking, this patient’s likelihood of reoperation increases from 6.29 to 6.72 (a deterioration of 6%).

## Discussion

We introduced the concept of HT management based on analysing the relationships between modifiable factors and the degree of medical risk. We have shown how the contributions of individual factors or their combinations that reduce future medical risk can be viewed in detail based on the patient’s condition. A significant advantage of this approach is the automated support through a CDMT, which offers alternative decisions and traces how the choice of an alternative creates an HT to reduce risks gradually.

This research focuses primarily on the possibilities of influencing the patient’s future concerning factors that provably affect their health and risk of disease [28]. Accordingly, the patients can influence, at least in part, some of the factors on their HT by deciding to change their behaviour and lifestyle. Therefore, the management of future HT should be dependent on a particular disease with growing participation from the patient. The CDMT serves to support the clinician’s and patient’s decisions. In our approach to HT management using FCA, we work only with specific risk and a set of binary factors, at least some of which can be influenced by a change in patient behaviour at particular time points in their life. Although the binarisation of factors may appear to be limiting, EHR factors are often inherently binary (positive versus negative) or can be easily and naturally binarised (such as non-smoker/ex-smoker versus smoker, BMI ≤ 30 versus BMI > 30). For more complex tasks, numerical factors and overlapping clustering methods can be used [21, 22]. Thanks to the visual hierarchical form, FCA provides well-explained and interpretable outcomes and enables the numerical calculation of an event’s probability of occurrence within a cluster [29, 30]. This allows the degree of risk to be easily quantified for different combinations of factors and proposes selective trajectories to reduce risk. A significant advantage of this approach is the use of overlapping clusters, thus providing patient(s) with more options to reduce their quantifiable risk and consider how to reduce their risk in the longer term. These features are not available using traditional methods, such as prediction in the meaning of classification or non-overlapping clustering.

This methodology was applied to a real-world dataset from orthopaedics, showing the influence of lifestyle factors on the risk of TKA reoperation in a cohort of 1885 patients from a registry of TKAs [17]. In this case, the patient’s condition is understood as a set of factors recorded in the register of TKAs (the EHR). At least some of the factors are assumed to be modifiable by the patient. Moreover, any combination of factors defines a group of patients as similar in terms of these factors. For each combination of selected factors, the degree of medical risk can be quantified, and combinations of these factors may overlap. As a target tool based on this approach, a user-friendly CDMT was created that implements the HT management model. Using the CDMT, the patient can make decisions about their short-term or long-term future, either by themselves or under the supervision of their clinician or physiotherapist. Thanks to the visualisation of one or more HT, decisions can be made with a longer-term expectation. The clinical relevance of the observed results and current orthopaedic opinion are discussed in detail in the Additional file 1. Importantly, this model may be adapted for local data derived from the hospital in which the patient will be operated on, thus establishing patient expectations based on local real-world patient data. We are aware that a CDMT based on data from other TKA/hospital registers may offer other results, as the parameters may be influenced by other factors, such as genetic background, lifestyle, environment and the local health care system, contributing to the patient outcome.

The presented model of HT management can be broadly applied. In the era of precision medicine and health, it is crucial to identify critical factors that significantly increase or reduce health risk(s) in all branches of medicine [10, 31]. The essential task, not just in orthopaedics, is to identify the best-suited therapy for an individual patient, as well as to minimise the harm associated with a particular intervention, because even well-established therapeutic interventions have been questioned in the last few decades [14, 32]. Several other examples in the literature identify risk factors for various diseases, such as diabetes [33, 34], cardiovascular disease [35, 36], breast [37, 38] and lung [39, 40] cancer and many others that are straightforwardly applicable to HT management, as shown in Table 2.

**Table 2:** Examples of possible uses for HT management

Disease	Modifiable negative factors
Diabetes [33, 34]	Being overweight or obese, physical inactivity, high blood pressure, high cholesterol, tobacco smoking, unhealthy eating, heavy alcohol consumption
Cardiovascular disease [35, 36]	Being overweight or obese, physical inactivity, unhealthy eating, alcohol consumption, smoking, high blood pressure, diabetes, sodium intake
Breast cancer [37, 38]	Long-term use of combination hormone replacement therapy (oestrogen–progestin), obesity, alcohol consumption, late pregnancy or never being pregnant, night-shift work, physical inactivity
Lung cancer [39, 40]	Smoking (cigarette, cigar and pipe), second-hand smoking, beta carotene supplements in heavy smokers, alcohol consumption, exposure to chemicals, air pollution
TKA reoperation [17]	BMI, smoking, low activity, no sports, no long-distance walking
Autoimmune diseases [47]	Being obese, smoking, unhealthy eating, physical inactivity, exposure to certain infections, certain medications, exposure to toxic agents

The advantage of this and other data-driven approaches is that if, for example, the impact of different factors on health varies in different regions, then HT management does not change, but HTs may differ. From this point of view, HTs must depend on (i) correct diagnosis (assessment of the patient’s condition), (ii) diagnosis made at the right place and at the right time (a point on an HT), (iii) intervention proposals or lifestyle changes based on precise data obtained through precision medicine and EHR maintenance and (iv) selecting a path to the next point on the trajectory that has a quantifiably lower risk. Showing the likelihood of the development of a complication when one or more factors are changed and estimating a patient’s medical risk can also significantly support a patient’s motivation to change potentially manageable factors [41].

The FCA method used in this study is also used as a cluster analysis method across many industries [19]. In the study of gene expression, FCA can be used to develop an efficient algorithm to find biologically important genes with a negative correlation [42]. Information retrieval, e-learning, expert systems and the semantic web often use FCA methods. In one of them [43], techniques based on different attribute-scaling methods are presented together with the concept of measuring cluster similarity and extending the method’s applicability by similarity reasoning. This extension is shown in the example of the automatic generation of ontologies using the identification of overlapping knowledge in the common domain. Additionally, the application of our theoretical model, for the first time, uses overlapping clustering using FCA to calculate the contribution of lifestyle factors to early reoperations and suggests the potential usefulness of FCA in HT management in clinical practices on real-world cohorts.

In the future, precision medicine will, thanks to new technologies, provide an increasing amount of precise patient data, which will be collected and stored in EHRs [11, 29]. By its nature, the data will offer a view of the patient over time. Due to the growing potential of large-scale data analysis, the relationships between patient data and the risks associated with various diseases will be detected promptly. The data stored in EHRs, whose informational value will increase, are highly heterogeneous, and their analysis will increasingly show how groups of factors describe the combined risks of different diseases [29, 44]. Therefore, it is also necessary to consider the degree of overlapping of similar patients’ groups that can be described by similar characteristics and common risks.

Furthermore, patients’ willingness to provide their data and be part of precision medicine research is increasing. It should, therefore, be evident that, in return for this willingness, patients should receive not only treatment or another type of intervention but also a forecast of how they can influence their future. Here, it is necessary to assume that there should be alternatives (modifiable factors) that will gradually reduce risks and are based on choices that are acceptable to the patient. Furthermore, the consistent detection of groups of patients with similar combinations of risks leads to overlaps, allowing us to think more comprehensively about which factors are worth influencing in terms of reducing risks. Here, lifestyle data is an excellent example, showing the patient how they can think about their future depending on precisely quantifiable information.

The approach presented has limitations. First, the behaviour of our theoretical model is shown on an example dataset from one TKA register. Of course, other examples and from different parts of medicine could be used to show the applicability of the model. In addition, future studies should prove the performance of the model for a particular data collected in EHR. Second, in relation to our example, preoperative factors, such as comorbidity, diabetes and allergies, were not evaluated because these patients were not significantly represented in this cohort: larger cohorts of patients will be required to perform such subanalyses. Third, the value of the model’s recommendations should be confirmed in a prospective long-term study comparing the model recommendations, their implementation with obtained outcomes of interest. Finally, we did not take into account that HT management also depends on a wide range of heterogeneous parameters, such as individual psychological traits, education, previous experience, knowledge, training and decision-making. Nevertheless, this model can help support decision-making in the near future after validation using different clinical topics in different clinical settings.

## Conclusions

This theoretical model of HT management using FCA was presented as working with specific risks and a set of negative/positive binary factors, at least some of which may be influenced by a change in patient behaviour. This allows the degree of medical risk for different combinations of risk factors to be quantified and alternative selective trajectories to be proposed to reduce (or enhance) this risk. Estimating a patient’s medical risk can significantly support a patient’s motivation to change potentially manageable factors by showing the likelihood of adverse effects when one or more factors are changed and suggesting possible ways to influence HT positively. The approach has broad applicability for HT management, irrespective of the branch of medicine. The usefulness of this theoretical model to change the personal risks of disease and adverse health effects should be proved using real-world EHR datasets in future studies.

## Description of used terms

*Precision medicine*: Proposes the tailoring of medical decisions and treatments to a subgroup of patients and takes individual variability, such as genes, environment and lifestyle, into account [45].

*Precision health*: Focuses on predicting and preventing diseases precisely and considers various factors that help maintain health throughout life [46]. The common goals are long-term health, reducing the risk of disease and minimising the impact of disease. From a long-term perspective, it is, therefore, a matter of finding a way to improve (or at least not significantly worse) the condition (i.e. the outcome) of a healthy person over time, and to minimise the risks associated with treatment and intervention for a patient.

## Supplementary Information

**Additional file 1: Table S1.** Patient records in the form of Formal context. **Table S2. ** List of all concepts for the context in Additional file 1: Table S1. **Figure S1. ** Concept lattice for the context in Additional file 1: Table S1. **Table S3. **Demographic and lifestyle parameters in the TKA cohort used for forecast model. TKA, total knee arthroplasty; yrs, years; BMI, Body Mass Index; VAS, Visual Analog Scale; KSS, Knee Society Score; UCLA, University of California Los Angeles; NA, not available. **Table S4. **Concepts in lifestyle parameters in the dataset of younger women. **Table S5. **Concepts in lifestyle parameters in the dataset of older women. **Table S6. **Concepts in lifestyle parameters in the dataset of younger men. **Table S7. **Concepts in lifestyle parameters in the dataset of older men. **Figure S2. **Sequences of concepts associated with a reduction of the likelihood of reoperation in TKA patient groups: A) younger women, B) older women, C) younger men, D) older men.

## Data Availability

The data and materials from this study are available from the corresponding author upon reasonable request.

## Abbreviations

BMI: Body mass index
CDMT: Clinical decision-making tool
CDSS: Clinical decision support system
EHR: Electronic health record
FCA: Formal concept analysis
HT: Health trajectory
TKA: Total knee arthroplasty
