# Supplementary material for: A theoretical model of health management using data-driven decision-making: the future of precision medicine and health
Source: J Transl Med. 2021 Feb 15;19:68. doi: 10.1186/s12967-021-02714-8 (PMC7885377; doi:10.1186/s12967-021-02714-8)
Supplement: Supplementary file 1 — Additional file 1: Table S1. Patient records in the form of Formal context. Table S2. List of all concepts for the context in Additional file 1: Table S1. Figure S1. Concept lattice for the context in Additional file 1: Table S1. Table S3. Demographic and lifestyle parameters in the TKA cohort used for forecast model. TKA, total knee arthroplasty; yrs, years; BMI, Body Mass Index; VAS, Visual Analog Scale; KSS, Knee Society Score; UCLA, University of California Los Angeles; NA, not available. Table S4. Concepts in lifestyle parameters in the dataset of younger women. Table S5. Concepts in lifestyle parameters in the dataset of older women. Table S6. Concepts in lifestyle parameters in the dataset of younger men. Table S7. Concepts in lifestyle parameters in the dataset of older men. Figure S2. Sequences of concepts associated with a reduction of the likelihood of reoperation in TKA patient groups: A) younger women, B) older women, C) younger men, D) older men. [file 12967_2021_2714_MOESM1_ESM.docx]

**Additional file 1:**

**A theoretical model of health management using data-driven decision-making: the future of precision medicine and health**

Eva Kriegova, Milos Kudelka, Martin Radvansky, Jiri Gallo

**Description of formal concept analysis (FCA)**

Formal concept analysis (1) is one of the methods used for the analysis of object-attributed data. This method can derive implicit relationships between sets of objects and a set of binary attributes from a formal context described in tabular form. The output of this method consists of a set of formal concepts and their hierarchical structure: a concept lattice.

Once given the data source in the form of tabular data, formal context $\mathbb{K}$ can be defined. It can be described as $\mathbb{K=(}G,M,I)$, which is a triplet consisting of a set of objects *G*, a set of attributes *M* and $I\subseteq G\times M$ is a binary relation between *G* and *M*.

A simple example of a formal context $\mathbb{K}$ is shown in Table S1. Each row is an object representing one patient, and each column is an attribute representing one factor.

**Table S1** Patient records in the form of Formal context.

|  | NoSmoking | LowBMI | Sport | Activity |
| --- | --- | --- | --- | --- |
| Patient 1 | × |  | × |  |
| Patient 2 |  | × |  | × |
| Patient 3 | × |  | × | × |
| Patient 4 |  | × | × |  |

For $A\subseteq G$, we define $A^{\uparrow}=\left\{ m\in M \right|g\in A:\left( g,m \right)\in I\}$, in the meaning that $A^{\uparrow}$ is a set of all attributes common to all objects of set *A.*

Dually for $B\subseteq M$, we define $B^{\downarrow}=\{g\in G|\forall m\in B:\left( g,m \right)\in I\}$ as a set of all objects that have all attributes of set *B*.

The formal concept of 𝕂 is a pair $(A,B)$ where $A\subseteq G, B\subseteq M$ with property $A^{\uparrow}=B and B^{\downarrow}=A$. The most important relationships between formal concepts are given by the sub-concept – super-concept relation. It forms the hierarchical structure of complete lattice so-called Galois (concept) lattice.

| **Table S2** List of all concepts for the context in Table S1   \| **#** \| **Formal concept** \| \| --- \| --- \| \| 1 \| { Patient 1, Patient 2, Patient 3, Patient 4 }  NO COMMON FACTORS \| \| 2 \| { Patient 1, Patient 3, Patient 4 }  { Sport } \| \| 3 \| { Patient 1, Patient 3 }  { NoSmoking, Sport } \| \| 4 \| { Patient 2, Patient 4 }  { LowBMI } \| \| 5 \| { Patient 2, Patient 3 }  { Activity } \| \| 6 \| { Patient 4 }  { LowBMI, Sport } \| \| 7 \| { Patient 3 }  { NoSmoking, Sport, Activity } \| \| 8 \| { Patient 2 }  { LowBMI, Activity } \| \| 9 \| NO PATIENTS  { NoSmoking, LowBMI, Sport, Activity } \| |
| --- | --- | --- | --- | --- | --- | --- | --- | --- | --- | --- | --- | --- | --- | --- | --- | --- | --- | --- | --- | --- |

BMI=Body Mass Index. “Positive” factors were as follows: Activity (Activity), Long distance walking (LongDistWalk), no-smoking (NoSmoking), sport (Sport), BMI<30 (lowBMI); no “positive” factors present (NO COMMON FACTORS).

Table S2 shows all of the formal concepts (patient groups) detected in the context of Table S1. The concept lattice in Figure S1 shows the structure of concepts ordered by the generalisation–specialisation relationship organised from top to bottom. The colours of individual concepts correspond to the number of patients in the individual concepts. The top concept (#1) is a group containing all four patients without any common attributes. The last group (#9) does not contain any patients because there are no patients with all common factors.

| 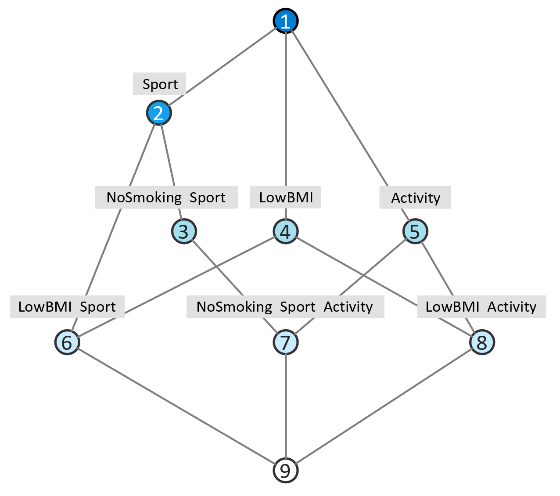  **Figure S1** Concept lattice for the context in Table S1  **Quantifying contributions of changes in modifiable clinical factors**  If investigated factors are modifiable, then sequences of concepts could be identified, in which: i) the factors in the preceding concept are contained in the succeeding concept of sequence, ii) patients in the succeeding concept are contained in the preceding concept of the sequence, iii) the empirical probability of an event in the succeeding concept is lower than in the preceding concept. The obtained sequences can then be used in a decision support system that can offer physicians and patients factors reducing the likelihood of the event, in our case, reoperation.  Nevertheless, some concepts contain very few patients and zero reoperations. The empirical probability of reoperation is zero in these cases; this is not reasonably usable information from the perspective of further analysis. Therefore, before further analysis was performed, we introduced some uncertainty into the dataset that slightly changed the empirical probability. In this modification, one extra patient was added to each concept (patient group) with the probability of reoperation corresponding to the total empirical probability $p_{D}$ of the entire dataset. The modified probability of reoperation in concept *C* is then $C=\frac{r_{C}+p_{D}}{n_{C}+1}$, where $n_{C}$ is the number of patients in concept *C*, and $r_{C}$ is the number of patients with reoperation in concept *C*. This modification does not change the reoperation probability for the entire dataset, for a higher empirical probability than total probability decreases, and for a lower than total probability increases the reoperation probability for the individual concepts. This is also true for the zero value that also increased the probability.  **Table S3. Demographic and lifestyle parameters in the TKA cohort used for forecast model.** TKA, total knee arthroplasty; yrs, years; BMI, Body Mass Index; VAS, Visual Analog Scale; KSS, Knee Society Score; UCLA, University of California Los Angeles; NA, not available.   \|  \|  \| Younger Females  (≤71 yrs) \| \| Older Females  (>71 yrs) \| \| Younger Males  (≤66 yrs) \| \| Older Males  (>66 yrs) \| \| \| --- \| --- \| --- \| --- \| --- \| --- \| --- \| --- \| --- \| --- \| \| Parameters \| Value/range \| Number \| % \| Number \| % \|  \|  \| Number \| % \| \| Gender \| Male \| 0 \| 0 \| 0 \| 0 \| 275 \| 39.6 \| 420 \| 60.4 \| \|  \| Female \| 670 \| 56.3 \| 520 \| 43.7 \| 0 \| 0 \| 0 \| 0 \| \| Age [years] \| ≤50 \| 11 \| 1.6 \| 0 \| 0.0 \| 8 \| 2.9 \| 0 \| 0.0 \| \|  \| 51–70 \| 598 \| 89.3 \| 0 \| 0.0 \| 267 \| 97.1 \| 131 \| 31.2 \| \|  \| 71–80 \| 61 \| 9.1 \| 451 \| 86.7 \| 0 \| 0.0 \| 259 \| 61.7 \| \|  \| >80 \| 0 \| 0.0 \| 69 \| 13.3 \| 0 \| 0.0 \| 30 \| 7.1 \| \| Weight [kg] \| ≤60 \| 18 \| 2.7 \| 34 \| 6.5 \| 3 \| 1.1 \| 1 \| 0.2 \| \|  \| 61–80 \| 218 \| 32.5 \| 278 \| 53.5 \| 30 \| 10.9 \| 101 \| 24.0 \| \|  \| 81–100 \| 301 \| 44.9 \| 181 \| 34.8 \| 118 \| 42.9 \| 218 \| 51.9 \| \|  \| >100 \| 133 \| 19.9 \| 27 \| 5.2 \| 124 \| 45.1 \| 100 \| 23.8 \| \| Height [cm] \| ≤157 \| 82 \| 12.2 \| 112 \| 21.5 \| 1 \| 0.4 \| 3 \| 0.7 \| \|  \| 158–171 \| 510 \| 76.1 \| 378 \| 72.7 \| 47 \| 17.1 \| 108 \| 25.7 \| \|  \| 172–184 \| 76 \| 11.3 \| 30 \| 5.8 \| 188 \| 68.4 \| 268 \| 63.8 \| \|  \| >184 \| 2 \| 0.3 \| 0 \| 0 \| 39 \| 14.2 \| 41 \| 9.8 \| \| BMI [kg/m^2^] \| ≤25 \| 61 \| 9.1 \| 74 \| 14.2 \| 21 \| 7.6 \| 50 \| 11.9 \| \|  \| 26–30 \| 174 \| 26.0 \| 187 \| 36.0 \| 95 \| 34.5 \| 187 \| 44.5 \| \|  \| 31–35 \| 226 \| 33.7 \| 178 \| 34.2 \| 89 \| 32.4 \| 147 \| 35.0 \| \|  \| >35 \| 209 \| 31.2 \| 81 \| 15.6 \| 70 \| 25.5 \| 36 \| 8.6 \| \| Waiting for TKA [months] \| NA \| 0 \| 0.0 \| 0 \| 0.0 \| 0 \| 0.0 \| 3 \| 0.7 \| \|  \| ≤3 \| 167 \| 24.9 \| 148 \| 28.5 \| 63 \| 22.9 \| 111 \| 26.4 \| \|  \| 3–6 \| 279 \| 41.6 \| 230 \| 44.2 \| 113 \| 41.1 \| 164 \| 39.0 \| \|  \| 6–12 \| 97 \| 14.5 \| 59 \| 11.3 \| 45 \| 16.4 \| 54 \| 12.9 \| \|  \| >12 \| 127 \| 19.0 \| 83 \| 16.0 \| 54 \| 19.6 \| 88 \| 21.0 \| \| Osteoarthritis \| Primary \| 109 \| 16.3 \| 72 \| 13.8 \| 81 \| 29.5 \| 62 \| 14.8 \| \|  \| Secondary \| 561 \| 83.7 \| 448 \| 86.2 \| 194 \| 70.5 \| 358 \| 85.2 \| \| Charlson comorbidity score \| 0–2 \| 547 \| 81.6 \| 394 \| 75.8 \| 234 \| 85.1 \| 327 \| 77.9 \| \|  \| 3–6 \| 77 \| 11.5 \| 81 \| 15.6 \| 31 \| 11.3 \| 65 \| 15.5 \| \|  \| >6 \| 46 \| 6.9 \| 45 \| 8.7 \| 10 \| 3.6 \| 28 \| 6.7 \| \| Smoking \| No \| 538 \| 80.3 \| 479 \| 92.1 \| 166 \| 60.4 \| 298 \| 71 \| \|  \| Stop \| 56 \| 8.4 \| 23 \| 4.4 \| 56 \| 20.4 \| 90 \| 21.4 \| \|  \| Yes \| 76 \| 11.3 \| 18 \| 3.5 \| 53 \| 19.3 \| 32 \| 7.6 \| \| Walking aids \| No \| 332 \| 49.6 \| 162 \| 31.2 \| 172 \| 62.5 \| 215 \| 51.2 \| \|  \| Yes \| 338 \| 50.4 \| 358 \| 68.8 \| 103 \| 37.5 \| 205 \| 48.8 \| \| Walking distance \| Up to 1 km \| 566 \| 84.5 \| 449 \| 86.3 \| 195 \| 70.9 \| 329 \| 78.3 \| \|  \| > 1 km \| 104 \| 15.5 \| 71 \| 13.7 \| 80 \| 29.1 \| 91 \| 21.7 \| \| Pain preoperative \| Mild \| 2 \| 0.3 \| 2 \| 0.4 \| 5 \| 1.8 \| 6 \| 1.4 \| \|  \| Moderate \| 479 \| 71.5 \| 326 \| 62.7 \| 205 \| 74.5 \| 315 \| 75.0 \| \|  \| Severe \| 188 \| 28.1 \| 191 \| 36.7 \| 64 \| 23.3 \| 99 \| 23.6 \| \|  \| Catastrophic \| 1 \| 0.1 \| 1 \| 0.2 \| 1 \| 0.4 \| 0 \| 0.0 \| \| Preoperative VAS \| Low (0–2) \| 1 \| 0.1 \| 1 \| 0.2 \| 2 \| 0.7 \| 4 \| 1 \| \|  \| Middle (3–5) \| 221 \| 33.0 \| 156 \| 30.0 \| 122 \| 44.4 \| 163 \| 38.8 \| \|  \| High (>5) \| 448 \| 66.9 \| 363 \| 69.8 \| 151 \| 54.9 \| 253 \| 60.2 \| \| Axis deviation \| None \| 183 \| 27.3 \| 116 \| 22.3 \| 63 \| 22.9 \| 86 \| 20.5 \| \|  \| Valgus \| 93 \| 13.9 \| 81 \| 15.6 \| 12 \| 4.4 \| 21 \| 5 \| \|  \| Varus \| 394 \| 58.8 \| 323 \| 62.1 \| 200 \| 72.7 \| 313 \| 74.5 \| \| Kellgren-Lawrence \| II–III C \| 474 \| 70.7 \| 342 \| 65.8 \| 194 \| 70.5 \| 281 \| 66.9 \| \|  \| III D–IV D \| 196 \| 29.3 \| 178 \| 34.2 \| 81 \| 29.5 \| 139 \| 33.1 \| \| KSS general \| ≤70 \| 627 \| 93.6 \| 492 \| 94.6 \| 250 \| 90.9 \| 368 \| 87.6 \| \|  \| 71–80 \| 37 \| 5.5 \| 24 \| 4.6 \| 22 \| 8.0 \| 46 \| 11.0 \| \|  \| >80 \| 6 \| 0.9 \| 4 \| 0.8 \| 3 \| 1.1 \| 6 \| 1.4 \| \| KSS functional \| ≤70 \| 635 \| 94.8 \| 500 \| 96.2 \| 227 \| 82.5 \| 366 \| 87.1 \| \|  \| >71 \| 35 \| 5.2 \| 20 \| 3.8 \| 48 \| 17.5 \| 54 \| 12.9 \| \| UCLA Activity \| No/low (1–3) \| 545 \| 81.3 \| 460 \| 88.5 \| 181 \| 65.8 \| 325 \| 77.4 \| \|  \| Middle (4–6) \| 125 \| 18.7 \| 60 \| 11.5 \| 93 \| 33.8 \| 95 \| 22.6 \| \|  \| High (7–10) \| 0 \| 0.0 \| 0 \| 0.0 \| 1 \| 0.4 \| 0 \| 0.0 \| \| Sport activity \| NA \| 20 \| 3.0 \| 13 \| 2.5 \| 10 \| 3.6 \| 22 \| 5.2 \| \|  \| No \| 607 \| 90.6 \| 483 \| 92.9 \| 216 \| 78.5 \| 342 \| 81.4 \| \|  \| Active before \| 29 \| 4.3 \| 18 \| 3.5 \| 40 \| 14.5 \| 45 \| 10.7 \| \|  \| Active \| 14 \| 2.1 \| 6 \| 1.2 \| 9 \| 3.3 \| 11 \| 2.6 \| \| Complication \| No \| 395 \| 59.0 \| 270 \| 51.9 \| 160 \| 58.2 \| 216 \| 51.4 \| \|  \| Yes \| 275 \| 41.0 \| 250 \| 48.1 \| 115 \| 41.8 \| 204 \| 48.6 \| \| Reoperation \| No \| 643 \| 96.0 \| 495 \| 95.2 \| 245 \| 89.1 \| 393 \| 93.6 \| \|  \| Yes \| 27 \| 4.0 \| 25 \| 4.8 \| 30 \| 10.9 \| 27 \| 6.4 \| |
| --- | --- | --- | --- | --- | --- | --- | --- | --- | --- | --- | --- | --- | --- | --- | --- | --- | --- | --- | --- | --- | --- | --- | --- | --- | --- | --- | --- | --- | --- | --- | --- | --- | --- | --- | --- | --- | --- | --- | --- | --- | --- | --- | --- | --- | --- | --- | --- | --- | --- | --- | --- | --- | --- | --- | --- | --- | --- | --- | --- | --- | --- | --- | --- | --- | --- | --- | --- | --- | --- | --- | --- | --- | --- | --- | --- | --- | --- | --- | --- | --- | --- | --- | --- | --- | --- | --- | --- | --- | --- | --- | --- | --- | --- | --- | --- | --- | --- | --- | --- | --- | --- | --- | --- | --- | --- | --- | --- | --- | --- | --- | --- | --- | --- | --- | --- | --- | --- | --- | --- | --- | --- | --- | --- | --- | --- | --- | --- | --- | --- | --- | --- | --- | --- | --- | --- | --- | --- | --- | --- | --- | --- | --- | --- | --- | --- | --- | --- | --- | --- | --- | --- | --- | --- | --- | --- | --- | --- | --- | --- | --- | --- | --- | --- | --- | --- | --- | --- | --- | --- | --- | --- | --- | --- | --- | --- | --- | --- | --- | --- | --- | --- | --- | --- | --- | --- | --- | --- | --- | --- | --- | --- | --- | --- | --- | --- | --- | --- | --- | --- | --- | --- | --- | --- | --- | --- | --- | --- | --- | --- | --- | --- | --- | --- | --- | --- | --- | --- | --- | --- | --- | --- | --- | --- | --- | --- | --- | --- | --- | --- | --- | --- | --- | --- | --- | --- | --- | --- | --- | --- | --- | --- | --- | --- | --- | --- | --- | --- | --- | --- | --- | --- | --- | --- | --- | --- | --- | --- | --- | --- | --- | --- | --- | --- | --- | --- | --- | --- | --- | --- | --- | --- | --- | --- | --- | --- | --- | --- | --- | --- | --- | --- | --- | --- | --- | --- | --- | --- | --- | --- | --- | --- | --- | --- | --- | --- | --- | --- | --- | --- | --- | --- | --- | --- | --- | --- | --- | --- | --- | --- | --- | --- | --- | --- | --- | --- | --- | --- | --- | --- | --- | --- | --- | --- | --- | --- | --- | --- | --- | --- | --- | --- | --- | --- | --- | --- | --- | --- | --- | --- | --- | --- | --- | --- | --- | --- | --- | --- | --- | --- | --- | --- | --- | --- | --- | --- | --- | --- | --- | --- | --- | --- | --- | --- | --- | --- | --- | --- | --- | --- | --- | --- | --- | --- | --- | --- | --- | --- | --- | --- | --- | --- | --- | --- | --- | --- | --- | --- | --- | --- | --- | --- | --- | --- | --- | --- | --- | --- | --- | --- | --- | --- | --- | --- | --- | --- | --- | --- | --- | --- | --- | --- | --- | --- | --- | --- | --- | --- | --- | --- | --- | --- | --- | --- | --- | --- | --- | --- | --- | --- | --- | --- | --- | --- | --- | --- | --- | --- | --- | --- | --- | --- | --- | --- | --- | --- | --- | --- | --- | --- | --- | --- | --- | --- | --- | --- | --- | --- | --- | --- | --- | --- | --- | --- | --- | --- | --- | --- | --- | --- | --- | --- | --- | --- | --- | --- | --- | --- | --- | --- | --- | --- | --- | --- | --- | --- | --- | --- | --- | --- | --- | --- | --- | --- | --- | --- | --- | --- | --- | --- | --- | --- | --- | --- | --- | --- | --- | --- | --- | --- | --- | --- | --- | --- | --- | --- | --- | --- | --- | --- | --- | --- | --- | --- | --- | --- | --- | --- | --- | --- | --- | --- | --- | --- | --- | --- | --- | --- | --- | --- | --- | --- | --- | --- | --- | --- | --- | --- | --- | --- | --- | --- | --- | --- | --- | --- | --- | --- | --- | --- | --- | --- | --- | --- | --- | --- | --- | --- | --- | --- | --- | --- | --- | --- | --- | --- | --- | --- | --- | --- | --- | --- | --- | --- | --- | --- | --- | --- | --- | --- | --- | --- | --- | --- | --- | --- | --- | --- | --- | --- | --- | --- | --- | --- | --- | --- | --- | --- | --- | --- | --- | --- | --- | --- | --- | --- | --- | --- | --- | --- | --- | --- | --- | --- | --- | --- | --- | --- | --- | --- | --- | --- | --- | --- | --- | --- | --- | --- | --- | --- | --- | --- | --- | --- | --- | --- | --- | --- | --- | --- | --- |

**Table S4. Concepts in lifestyle parameters in the dataset of younger women.**

| COMBINATION OF FACTORS | PATIENTS | REOPER. | PATIENTS | REOPER. | PROB. | UNCERT. |
| --- | --- | --- | --- | --- | --- | --- |
| NO COMMON FACTORS | 615 | 25 | 100·00% | 100·00% | 4·07% | 4·07% |
| LongDistWalk | 99 | 2 | 16·10% | 8·00% | 2·02% | 2·04% |
| Activity LowBMI | 54 | 1 | 8·78% | 4·00% | 1·85% | 1·89% |
| Activity LowBMI NoSmoking | 50 | 1 | 8·13% | 4·00% | 2·00% | 2·04% |
| LongDistWalk LowBMI | 48 | 0 | 7·80% | 0·00% | 0·00% | 0·08% |
| LongDistWalk LowBMI NoSmoking | 43 | 0 | 6·99% | 0·00% | 0·00% | 0·09% |
| Activity LongDistWalk LowBMI | 29 | 0 | 4·72% | 0·00% | 0·00% | 0·14% |
| Activity LongDistWalk LowBMI NoSmoking | 27 | 0 | 4·39% | 0·00% | 0·00% | 0·15% |
| Sport | 15 | 0 | 2·44% | 0·00% | 0·00% | 0·25% |
| NoSmoking Sport | 13 | 0 | 2·11% | 0·00% | 0·00% | 0·29% |
| LowBMI Sport | 12 | 0 | 1·95% | 0·00% | 0·00% | 0·31% |
| LowBMI NoSmoking Sport | 10 | 0 | 1·63% | 0·00% | 0·00% | 0·37% |
| LongDistWalk Sport | 5 | 0 | 0·81% | 0·00% | 0·00% | 0·68% |
| Activity Sport | 5 | 0 | 0·81% | 0·00% | 0·00% | 0·68% |
| Activity LowBMI Sport | 4 | 0 | 0·65% | 0·00% | 0·00% | 0·81% |
| LongDistWalk LowBMI Sport | 4 | 0 | 0·65% | 0·00% | 0·00% | 0·81% |
| Activity NoSmoking Sport | 4 | 0 | 0·65% | 0·00% | 0·00% | 0·81% |
| LongDistWalk NoSmoking Sport | 4 | 0 | 0·65% | 0·00% | 0·00% | 0·81% |
| LongDistWalk LowBMI NoSmoking Sport | 3 | 0 | 0·49% | 0·00% | 0·00% | 1·02% |
| Activity LowBMI NoSmoking Sport | 3 | 0 | 0·49% | 0·00% | 0·00% | 1·02% |
| Activity LongDistWalk NoSmoking Sport | 2 | 0 | 0·33% | 0·00% | 0·00% | 1·36% |
| Activity LongDistWalk LowBMI NoSmoking Sport | 1 | 0 | 0·16% | 0·00% | 0·00% | 2·03% |

Legend: In the “PATIENTS” column, there is the number of patients in the concept. In the “REOPER.” column, there is the number of patients who had to be reoperated early. The last column shows the percentage of probabilities, including the empirical probability of reoperation in the concept (“PROB.” column). The values in the “UNCERT.” means uncertainty introduced into the dataset probability. BMI=Body Mass Index. “Positive” factors were as follows: Activity (Activity), Long distance walking (LongDistWalk), no-smoking (NoSmoking), sport (Sport), BMI<30 (lowBMI); no “positive” factors present (NO COMMON FACTORS).

**Table S5. Concepts in lifestyle parameters in the dataset of older women.**

| COMBINATION OF FACTORS | PATIENTS | REOPER. | PATIENTS | REOPER. | PROB. | UNCERT. |
| --- | --- | --- | --- | --- | --- | --- |
| NO COMMON FACTORS | 502 | 25 | 100·00% | 100·00% | 4·98% | 4·98% |
| NoSmoking | 489 | 24 | 97·41% | 96·00% | 4·91% | 4·91% |
| LowBMI | 252 | 16 | 50·20% | 64·00% | 6·35% | 6·34% |
| LowBMI NoSmoking | 241 | 15 | 48·01% | 60·00% | 6·22% | 6·22% |
| LongDistWalk | 67 | 2 | 13·35% | 8·00% | 2·99% | 3·01% |
| LongDistWalk NoSmoking | 65 | 2 | 12·95% | 8·00% | 3·08% | 3·11% |
| Activity | 59 | 2 | 11·75% | 8·00% | 3·39% | 3·42% |
| Activity NoSmoking | 58 | 2 | 11·55% | 8·00% | 3·45% | 3·47% |
| LongDistWalk LowBMI NoSmoking | 43 | 2 | 8·57% | 8·00% | 4·65% | 4·66% |
| Activity LowBMI NoSmoking | 35 | 1 | 6·97% | 4·00% | 2·86% | 2·92% |
| Activity LongDistWalk | 26 | 0 | 5·18% | 0·00% | 0·00% | 0·18% |
| Activity LongDistWalk NoSmoking | 25 | 0 | 4·98% | 0·00% | 0·00% | 0·19% |
| Activity LongDistWalk LowBMI NoSmoking | 16 | 0 | 3·19% | 0·00% | 0·00% | 0·29% |
| NoSmoking Sport | 7 | 0 | 1·39% | 0·00% | 0·00% | 0·62% |
| LowBMI NoSmoking Sport | 5 | 0 | 1·00% | 0·00% | 0·00% | 0·83% |
| LongDistWalk NoSmoking Sport | 5 | 0 | 1·00% | 0·00% | 0·00% | 0·83% |
| Activity LongDistWalk NoSmoking Sport | 4 | 0 | 0·80% | 0·00% | 0·00% | 1·00% |
| Activity LongDistWalk LowBMI NoSmoking Sport | 3 | 0 | 0·60% | 0·00% | 0·00% | 1·25% |

Legend: In the “PATIENTS” column, there is the number of patients in the concept. In the “REOPER.” column, there is the number of patients who had to be reoperated early. The last column shows the percentage of probabilities, including the empirical probability of reoperation in the concept (“PROB.” column). The values in the “UNCERT.” means uncertainty introduced into the dataset probability. BMI=Body Mass Index. “Positive” factors were as follows: Activity (Activity), Long distance walking (LongDistWalk), no-smoking (NoSmoking), sport (Sport), BMI<30 (lowBMI); no “positive” factors present (NO COMMON FACTORS).

**Table S6. Concepts in lifestyle parameters in the dataset of younger men.**

| COMBINATION OF FACTORS | PATIENTS | REOPER. | PATIENTS | REOPER. | PROB. | UNCERT. |
| --- | --- | --- | --- | --- | --- | --- |
| NO COMMON FACTORS | 245 | 28 | 100·00% | 100·00% | 11·43% | 11·43% |
| NoSmoking | 213 | 26 | 86·94% | 92·86% | 12·21% | 12·20% |
| LowBMI | 113 | 15 | 46·12% | 53·57% | 13·27% | 13·26% |
| Activity | 92 | 12 | 37·55% | 42·86% | 13·04% | 13·03% |
| LowBMI NoSmoking | 90 | 15 | 36·73% | 53·57% | 16·67% | 16·61% |
| LongDistWalk | 78 | 8 | 31·84% | 28·57% | 10·26% | 10·27% |
| Activity NoSmoking | 76 | 10 | 31·02% | 35·71% | 13·16% | 13·14% |
| LongDistWalk NoSmoking | 66 | 8 | 26·94% | 28·57% | 12·12% | 12·11% |
| Activity LongDistWalk | 52 | 7 | 21·22% | 25·00% | 13·46% | 13·42% |
| Activity LongDistWalk NoSmoking | 44 | 7 | 17·96% | 25·00% | 15·91% | 15·81% |
| Activity LowBMI | 42 | 7 | 17·14% | 25·00% | 16·67% | 16·54% |
| LongDistWalk LowBMI | 37 | 5 | 15·10% | 17·86% | 13·51% | 13·46% |
| Activity LowBMI NoSmoking | 33 | 7 | 13·47% | 25·00% | 21·21% | 20·92% |
| LongDistWalk LowBMI NoSmoking | 30 | 5 | 12·24% | 17·86% | 16·67% | 16·50% |
| Activity LongDistWalk LowBMI | 25 | 4 | 10·20% | 14·29% | 16·00% | 15·82% |
| Activity LongDistWalk LowBMI NoSmoking | 20 | 4 | 8·16% | 14·29% | 20·00% | 19·59% |
| Sport | 9 | 2 | 3·67% | 7·14% | 22·22% | 21·14% |
| Activity Sport | 7 | 1 | 2·86% | 3·57% | 14·29% | 13·93% |
| Activity NoSmoking Sport | 5 | 1 | 2·04% | 3·57% | 20·00% | 18·57% |
| LowBMI NoSmoking Sport | 4 | 1 | 1·63% | 3·57% | 25·00% | 22·29% |
| Activity LongDistWalk NoSmoking Sport | 3 | 0 | 1·22% | 0·00% | 0·00% | 2·86% |
| Activity LongDistWalk LowBMI NoSmoking Sport | 1 | 0 | 0·41% | 0·00% | 0·00% | 5·71% |

Legend: In the “PATIENTS” column, there is the number of patients in the concept. In the “REOPER.” column, there is the number of patients who had to be reoperated early. The last column shows the percentage of probabilities, including the empirical probability of reoperation in the concept (“PROB.” column). The values in the “UNCERT.” means uncertainty introduced into the dataset probability. BMI=Body Mass Index. “Positive” factors were as follows: Activity (Activity), Long distance walking (LongDistWalk), no-smoking (NoSmoking), sport (Sport), BMI<30 (lowBMI); no “positive” factors present (NO COMMON FACTORS).

**Table S7. Concepts in lifestyle parameters in the dataset of older men.**

| COMBINATION OF FACTORS | PATIENTS | REOPER. | PATIENTS | REOPER. | PROB. | UNCERT. |
| --- | --- | --- | --- | --- | --- | --- |
| NO COMMON FACTORS | 387 | 26 | 100·00% | 100·00% | 6·72% | 6·72% |
| NoSmoking | 366 | 23 | 94·57% | 88·46% | 6·28% | 6·29% |
| LowBMI | 222 | 15 | 57·36% | 57·69% | 6·76% | 6·76% |
| LowBMI NoSmoking | 203 | 12 | 52·45% | 46·15% | 5·91% | 5·92% |
| Activity | 89 | 7 | 23·00% | 26·92% | 7·87% | 7·85% |
| Activity NoSmoking | 83 | 6 | 21·45% | 23·08% | 7·23% | 7·22% |
| LongDistWalk | 79 | 5 | 20·41% | 19·23% | 6·33% | 6·33% |
| LongDistWalk NoSmoking | 75 | 4 | 19·38% | 15·38% | 5·33% | 5·35% |
| Activity LowBMI | 56 | 4 | 14·47% | 15·38% | 7·14% | 7·14% |
| Activity LowBMI NoSmoking | 52 | 3 | 13·44% | 11·54% | 5·77% | 5·79% |
| LongDistWalk LowBMI | 50 | 3 | 12·92% | 11·54% | 6·00% | 6·01% |
| LongDistWalk LowBMI NoSmoking | 47 | 2 | 12·14% | 7·69% | 4·26% | 4·31% |
| Activity LongDistWalk | 47 | 5 | 12·14% | 19·23% | 10·64% | 10·56% |
| Activity LongDistWalk NoSmoking | 43 | 4 | 11·11% | 15·38% | 9·30% | 9·24% |
| Activity LongDistWalk LowBMI | 28 | 3 | 7·24% | 11·54% | 10·71% | 10·58% |
| Activity LongDistWalk LowBMI NoSmoking | 25 | 2 | 6·46% | 7·69% | 8·00% | 7·95% |
| NoSmoking Sport | 17 | 0 | 4·39% | 0·00% | 0·00% | 0·37% |
| LowBMI NoSmoking Sport | 12 | 0 | 3·10% | 0·00% | 0·00% | 0·52% |
| LongDistWalk NoSmoking Sport | 5 | 0 | 1·29% | 0·00% | 0·00% | 1·12% |
| Activity LongDistWalk NoSmoking Sport | 4 | 0 | 1·03% | 0·00% | 0·00% | 1·34% |
| LongDistWalk LowBMI NoSmoking Sport | 4 | 0 | 1·03% | 0·00% | 0·00% | 1·34% |
| Activity LongDistWalk LowBMI NoSmoking Sport | 3 | 0 | 0·78% | 0·00% | 0·00% | 1·68% |

Legend: In the “PATIENTS” column, there is the number of patients in the concept. In the “REOPER.” column, there is the number of patients who had to be reoperated early. The last column shows the percentage of probabilities, including the empirical probability of reoperation in the concept (“PROB.” column). The values in the “UNCERT.” means uncertainty introduced into the dataset probability. BMI=Body Mass Index. “Positive” factors were as follows: Activity (Activity), Long distance walking (LongDistWalk), no-smoking (NoSmoking), sport (Sport), BMI<30 (lowBMI); no “positive” factors present (NO COMMON FACTORS).

**Figure S2. Sequences of concepts associated with a reduction of the likelihood of reoperation in TKA patient groups:** A) younger women, B) older women, C) younger men, D) older men.

The edge (arrow) strength and its label correspond to the reduction of the risk of reoperation after adding a factor (percentage how much the risk of reoperation would be reduced). The same holds for vertex labels with factors and the numbers of patients. The ways of reduction of the likelihood of reoperation for our examples of older women and men is coloured in light green, the best-proposed way in dark green. “Positive” factors were as follows: Activity (Activity), Long distance walking (LongDistWalk), no-smoking (NoSmoking), BMI<30 (lowBMI); no “positive” factors present (NO COMMON FACTORS).


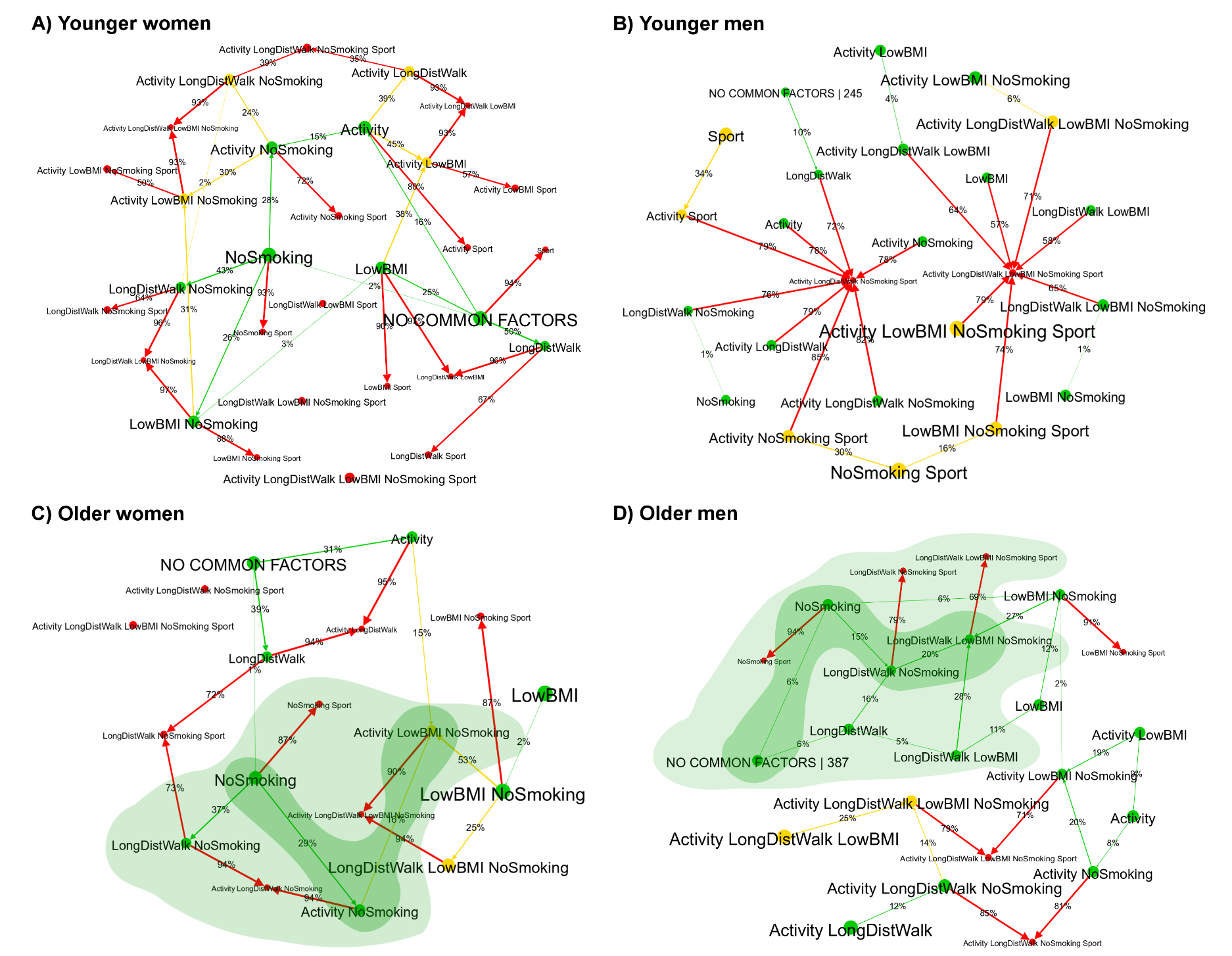


**The clinical meaning of the observed relationship between lifestyle factors and the risk of reoperations**

We tested our approach on a real-world cohort of patients who underwent TKA, where the information about early reoperations was derived from the patient registry. There is already a growing body of evidence that preoperative physical activity, BMI, and smoking affect the outcome of TKA (2-5), when excluding the influence of the surgeon, and other factors contributing to the risk of reoperation (6). For the first time, our forecast model enables us to quantify the contribution of these parameters to reoperations. Importantly, this model may be adopted for data derived from the hospital in which the patient will be operated. Additionally, our approach may show the reducing effect of changes in a particular factor(s) needed to advise patients on how to lower their individual risk of reoperation.

Our proof-of-principle modelling was performed in subgroups of younger and older men and women as different reoperation rates were reported in these groups (5, 6). In our cohort, the women markedly profited from physical activity with regard to lowering reoperation rates, which was not evident in men. The explanation for this is not easy since both gender and all age groups should benefit from regular physical activity (7, 8). However, our data is supported by a recent study with 21-year follow-up reporting that women participating in outdoor and leisure activities were significantly associated with long-term survival even when controlling for demographics, health and psychosocial variables, which was not observed for men (9). The negative association between activity and survival in older men might be linked to the different type, intensity, frequency and duration of activities between men and women reporting an active lifestyle. Sedentary behaviour is also much more pronounced in older men than women (10). However, there are concerns about the relevance of the data obtained from questionnaires on physical activity in terms of frequency, duration or intensity (11). Furthermore, there is a distinction between patients with less advanced osteoarthritis and those shortly before TKA, particularly in terms of their ability to participate in an exercise, resistance training or even stretching (12), with the latter patient group having much less room for improvement.

BMI also affects the outcomes of TKA in terms of satisfaction (13), complications (14) and the revision rate (15). Our study confirmed BMI as an important factor associated with early reoperations, particularly in women, less in men (6). Maintaining a normal weight is an advantage for all patients undergoing TKA as these patients are considered to be generally healthier compared to those with increased BMI. Therefore, non-obese people have an increased probability of uncomplicated wound healing, a lower probability of infection complications and lower morbidity and mortality after TKA (13, 14). As a result, similar to physical activity, a weight management plan and/or an exercise programme to work towards weight goals should already be addressed in less advanced stages of disease. We also found benefits in the contribution of patients’ history of sports activities and the ability to walk long distances. The positive influence of sport in a patient’s history on the reoperation rate was evident mainly in women and older men (6). This observation corresponds partly with the findings that older men who engage in strenuous sports (running, swimming, tennis, etc.) and those who increase their walking speed over time also have a lower risk of all-cause and some cause-specific mortality (16). Other studies reported the health relevance of leisure-time physical activity in both women and men (17).

Smoking is another known factor affecting infection (18), wound healing complications and reoperation rates in TKA patients (3). Our model confirmed that smoking is predominantly associated with reoperations in men. The benefit of the interruption of smoking is proven (3). The patient may become more motivated to stop smoking by the quantification and numerical expression of lowering their individual probability of reoperation as a result, and our approach may help motivate patients.

Although the main aim of this study was to demonstrate the utility of the mathematics behind patient data rather than to emphasise the clinical task this model should resolve, we need to mention some limitations. Among them are i) the inaccuracies associated with the collection of patient’s subjective information (physical activity data, sport); ii) some patients could seek care in another facility; however, the likelihood of this is very low due to the rules of the organisation of postoperative care in our country; iii) some patients may undergo surgery earlier than others as a consequence of the surgeon’s preference.

We did not validate the observations resulting from our example cohort as a prospective long-term study enrolling new patients who are suggested for a TKA reoperation is needed. Moreover, our approach assumes that individuals will be involved in data sharing, and a long-term follow-up implicates the suggested changes of modifiable factors to evaluate their influence on personal health trajectories (HT). However, regardless of these weaknesses, this approach, for the first time, enabled us to calculate the contribution of lifestyle factors to early reoperations after TKA and suggested the potential usefulness for HT management in clinical practice on a real-world cohort. HT management based on continuous data-driven decision-making is a long-term strategy for precision medicine and health, irrespective of the branch of medicine.

References

1. Ganter B, Wille, R. Formal Concept Analysis: Mathematical Foundations. Berlin: Springer - Verlag; 1999.

2. Kee JR, Mears SC, Edwards PK, Barnes CL. Modifiable Risk Factors Are Common in Early Revision Hip and Knee Arthroplasty. The Journal of arthroplasty. 2017;32(12):3689-92.

3. Matharu GS, Mouchti S, Twigg S, Delmestri A, Murray DW, Judge A, et al. The effect of smoking on outcomes following primary total hip and knee arthroplasty: a population-based cohort study of 117,024 patients. Acta orthopaedica. 2019;90(6):559-67.

4. Sezgin EA, A WD, Lidgren L, Robertsson O. Weight and height separated provide better understanding than BMI on the risk of revision after total knee arthroplasty: report of 107,228 primary total knee arthroplasties from the Swedish Knee Arthroplasty Register 2009-2017. Acta orthopaedica. 2019:1-4.

5. Bayliss LE, Culliford D, Monk AP, Glyn-Jones S, Prieto-Alhambra D, Judge A, et al. The effect of patient age at intervention on risk of implant revision after total replacement of the hip or knee: a population-based cohort study. Lancet. 2017;389(10077):1424-30.

6. Gallo J, Kriegova E, Kudelka M, Lostak J, Radvansky M. Gender Differences in Contribution of Smoking, Low Physical Activity, and High BMI to Increased Risk of Early Reoperation After TKA. J Arthroplasty. 2020;35(6):1545-57.

7. Ekelund U, Steene-Johannessen J, Brown WJ, Fagerland MW, Owen N, Powell KE, et al. Does physical activity attenuate, or even eliminate, the detrimental association of sitting time with mortality? A harmonised meta-analysis of data from more than 1 million men and women. Lancet. 2016;388(10051):1302-10.

8. Ekelund U, Tarp J, Steene-Johannessen J, Hansen BH, Jefferis B, Fagerland MW, et al. Dose-response associations between accelerometry measured physical activity and sedentary time and all cause mortality: systematic review and harmonised meta-analysis. BMJ. 2019;366:l4570.

9. Wasim A. F, O., Bath, P.A. Physical activity and long-term survival in older men and women: A 21 year longitudinal study. Activities, Adaptation & Aging. 2018;43(4):294-314.

10. Ashe MC, Michalowski VI, Chudyk AM, Gerstorf D, Madden KM, Hoppmann CA. Linked Lives: Exploring Gender and Sedentary Behaviors in Older Adult Couples. J Appl Gerontol. 2019:733464819868060.

11. de la Camara MA, Higueras-Fresnillo S, Cabanas-Sanchez V, Sadarangani KP, Martinez-Gomez D, Veiga OL. Criterion Validity of the Sedentary Behavior Question From the Global Physical Activity Questionnaire in Older Adults. J Phys Act Health. 2019:1-11.

12. Alrushud AS, Rushton AB, Kanavaki AM, Greig CA. Effect of physical activity and dietary restriction interventions on weight loss and the musculoskeletal function of overweight and obese older adults with knee osteoarthritis: a systematic review and mixed method data synthesis. BMJ Open. 2017;7(6):e014537.

13. Giesinger JM, Loth FL, MacDonald DJ, Giesinger K, Patton JT, Simpson A, et al. Patient-reported outcome metrics following total knee arthroplasty are influenced differently by patients' body mass index. Knee surgery, sports traumatology, arthroscopy : official journal of the ESSKA. 2018;26(11):3257-64.

14. Christensen TC, Wagner ER, Harmsen WS, Schleck CD, Berry DJ. Effect of Physical Parameters on Outcomes of Total Knee Arthroplasty. J Bone Joint Surg Am. 2018;100(21):1829-37.

15. Wagner ER, Kamath AF, Fruth K, Harmsen WS, Berry DJ. Effect of Body Mass Index on Reoperation and Complications After Total Knee Arthroplasty. J Bone Joint Surg Am. 2016;98(24):2052-60.

16. Hsu B, Merom D, Blyth FM, Naganathan V, Hirani V, Le Couteur DG, et al. Total Physical Activity, Exercise Intensity, and Walking Speed as Predictors of All-Cause and Cause-Specific Mortality Over 7 Years in Older Men: The Concord Health and Aging in Men Project. J Am Med Dir Assoc. 2018;19(3):216-22.

17. Wanner M, Tarnutzer S, Martin BW, Braun J, Rohrmann S, Bopp M, et al. Impact of different domains of physical activity on cause-specific mortality: a longitudinal study. Prev Med. 2014;62:89-95.

18. Nolan MB, Martin DP, Thompson R, Schroeder DR, Hanson AC, Warner DO. Association Between Smoking Status, Preoperative Exhaled Carbon Monoxide Levels, and Postoperative Surgical Site Infection in Patients Undergoing Elective Surgery. JAMA Surg. 2017;152(5):476-83.
